# Treatment of Asiatic Cholera

**Published:** 1848-05

**Authors:** 


*Treatment of Asiatic Cholera.*—The following remarks on the subject are extracted from a paper by M. Bally, whose name is attached to the most important therapeutical researches of the French school, and whose long career has been devoted to the study of epidemic maladies; his first appearance in the medical world was marked by the observation of the yellow fever at San Domingo, where the army, sent by the republic to that island was decimated by the scourge. At a later period, M. Bally again had an opportunity of witnessing a fearful attack of this disease in Spain. M. Bally collected carefully all the cases of cholera which were admitted into his wards at the Hötel Dieu during the epidemic of 1832, and he at present publishes the results of his meditations on the subject:—

1. The serum of the blood escapes from the digestive tube, and the brain, deprived of its habitual amount of stimulus, accomplishes its functions with great difficulty.

2. The eye is dried up from the arrest of the lachrymal secretions. The conjunctiva loses its brilliancy, and the nasal cavities are dessicated.

3. The mechanical part of respiration continues, but the vital and chemical functions of hematosis is in a great measure interrupted; hence the absence of oxydation of the blood, the disappearance of the scarlet colour, and of the cessation of production of animal heat.

11. The functions of the liver are interrupted. The gland retains even the quantity of bile previously secreted. The disease does not, therefore, deserve the name of "cholera."

12. It is more proper to call it "lymphatic choladrea," or "hydrocholadrea,," *i. e.* discharge of serum from the intestines.

13. The permeability of the integument is abolished, and also its function of exhalation; hence contact with the skin of a patient cannot communicate the disease. . . . . . .

The bladder is empty, the renal secretion having ceased. The mouth is parched; the salivary glands do not perform their function; hence an unquenchable thirst. . . . .

17. Digestion is annihilated. . . . .

19. But the intestine is endowed by the disease with new properties—with new functions.

20. The intestine becomes the only active organ.

21. Its secretion consists in the fluid portion of the blood. This opinion is not that of Professor Andral, but the difference of opinion may arise from the experiments of the professor having exclusively borne upon sporadic cases. . . . .

25. No efficient method of treatment has yet been adopted, and it is not the fault of medical practitioners: the cause of the inertness of treatment residing in the abolition of the absorbing powers of the intestine.

26. In countries where no treatment was employed, one half of the patients recovered; and in the countries where the patients were submitted to the most rational methods, one half died, often more, seldom less. . . . .

38. A poison circulates in the blood; an antidote must be sought for. That antidote cannot penetrate into the system through the intestinal surface; it must be, therefore, introduced through the respiratory organs, with the assistance of some of those marvellous anæsthetic agents, which modern science has placed at our disposal.—*Med. Times from Journal des Connais. Méd. Ch.*

